# Biocidal activity of polylactic acid-based nano-formulated abamectin on *Acyrthosiphon pisum* (Hemiptera: Aphididae) and the aphid predator *Adalia bipunctata* (Coleoptera: Coccinellidae)

**DOI:** 10.1371/journal.pone.0228817

**Published:** 2020-02-07

**Authors:** Changjiao Sun, Manli Yu, Zhanghua Zeng, Frédéric Francis, Haixin Cui, François Verheggen

**Affiliations:** 1 Gembloux Agro-Bio Tech, University of Liege, Gembloux, Belgium; 2 Institute of Environment and Sustainable Development in Agriculture, Chinese Academy of Agricultural Sciences, Beijing, China; University of Catania, ITALY

## Abstract

Abamectin is a common biocide used to control agricultural insect pests. However, the water insolubility of abamectin may result in extra organic solvent introduced in the environment. To solve this issue, it is desirable to develop nanoformulations to encapsulate abamectin with environment-friendly polymers. In this study, two polylactic acid based abamectin nanoformulations were prepared. The average particle sizes, measured by dynamic light scattering and transmission electron microscope, were 240 nm and 150 nm, respectively. The insecticidal activity of these nano-formulated abamectin was examined in the laboratory on the pea aphid, *Acyrthosiphon pisum* (Hemiptera: Aphididae). The acute toxicity of nano-formulated abamectin on non-target aphid predator *Adalia bipunctata* (Coleoptera: Coccinellidae) was also evaluated by topical, residual and oral exposure. The two nano-formulated abamectin had comparable insecticidal effect with commercial abamectin formulation against the pea aphid. Taking median lethal concentration (LC_50_) as the toxicological endpoint, nanoformulations had higher contact toxicity and lower oral toxicity to first-instar larvae of the predator *A*. *bipunctata*. These results are expected to contribute to the application of solvent-free nano-formulated pesticides that comply with the integrated pest management (IPM) strategies.

## Introduction

One of the global challenges faced by the agriculture industry is the sustainable food production for the rapidly growing human population, reaching 9.7 billion of individuals by 2050 [1–2]. Therefore, plant protection products and fertilizers are required to maximize the agricultural productivity [3]. In order to avoid the well-documented deleterious effects of pesticides, the efforts of agrochemical industry are not only focused on looking for new active substances, but also in new pesticide formulations [4].

During the last two decades, nanotechnology has been considered to have the potential to lead revolution in agricultural practices, especially in agrochemicals [5–6]. The development of nanotechnologies applied to pesticide formulations has facilitated the safe application of conventional pesticides by achieving precise and targeted delivery [7–9]. Besides, nanopesticide formulations may decrease the use of organic solvent and improve the biological efficacy of pesticides owing to the increasing dispersity, wettability, and penetration properties [6, 10]. Among all the nanopesticides, polymer-based nanoformulations are regarded as having the greatest potential for further development and practical application, due to their biocompatibility, biodegradability, modifiability and miscibility properties [10–13].

Avermectins represent a class of macrocyclic lactones that are produced by the soil dwelling actinomycete, *Streptomyces avermitilis* [14]. There are eight structural components, and abamectin is a mixture of avermectin B_1a_ and avermectin B_1b_. With a broad spectrum of activity, abamectin is one of most used biocides worldwide to control agricultural pests for its insecticide and acaricide activities [15]. However, the water insolubility of abamectin may result in extra organic solvent introduced in the environment [16], and abamectin is also susceptible to ultraviolet and strong acidic or alkaline conditions, which may cause premature degradation [17]. A great deal of efforts has been made to provide protection and sustainable release of abamectin, among which developing nanoformulations to encapsulate abamectin with environment-friendly polymers is an effective strategy [18].

Polylactic acid (PLA) is a USFDA-approved polymeric material that is widely employed as drug or cell carrier in the medical area for its biodegradable and mechanical properties that can be modified [19]. Active ingredients (AI) could be protected from photodegradation after encapsulation by PLA nanoparticles [15, 20]. Tannic acid (TA) is a plant polyphenol that exhibits antioxidant, antibacterial, antimicrobial, antimutagenic, and anticarcinogenic properties [21]. The unique structural properties of TA facilitate the interaction with other materials via electrostatic, hydrogen bonding, and hydrophobic interactions [21]. Due to the adhesive properties, TA-modified compounds were employed as coating materials and used for control of bacterial and mammalian cell adhesion, radical scavenging and marine fouling [22–24]. Therefore, TA modified nanopesticide could improve the adhesion of AI on target crops and pests. Besides, TA could also enhance the dispersibility and biocompatibility of nanomaterials [25], which may result in better efficacy of TA-modified nanopesticides.

Naturally-occurring biological control agents, such as arthropod predators, could control pests such as aphids in a wide range of cropping systems. However, the application of insecticide may disrupt the biological services through lethal and sublethal effects, including suppressing the population growth, extending the pre-adult period, and altering the mRNA expression in the progeny generation [26–29]. Nanopesticides may behave differently from conventional pesticides, so it is necessary to evaluate the effects on pest and non-target organisms [30].

In this study laboratory bioassays were conducted to identify the insecticidal effect of two nano-formulated abamectin on a major aphid species, *Acyrthosiphon pisum* (Hemiptera: Aphididae). *Acyrthosiphon pisum* is a worldwide distributed pest and a vector of more than 30 virus diseases [31]. In addition, as the protection of natural enemies of aphids in agricultural habitats remains an imperative issue, potential adverse effects on the non-target aphid predators *Adalia bipunctata* (Coleoptera: Coccinellidae) were also evaluated.

## Materials and methods

### Chemicals

Abamectin at a purity of 95.6% (pure technical grade) was purchased from Qilu Pharmaceutical Company, Ltd., China. PLA (molecular weight of 100,000) was purchased from Daigang Biomaterial Company, China. Poly(vinyl alcohol) (PVA) and agar were purchased from Sigma Aldrich. Dichloromethane (CH_2_Cl_2_, 99.8%), TA (95%), poly(ethylene glycol) (PEG, molecular weight of 10,000) were purchased from Bailingwei Technology Company, Ltd., China. A commercial emulsifiable concentrate (EC) containing 18 g/L of abamectin (Vertimec) was obtained from Syngenta, Belgium.

### Preparation of nano-formulated abamectin

PLA-based nano-formulated abamectin was synthesized according to a previously reported emulsion solvent evaporation method (O/W) [32]. PLA (40 mg/mL) and abamectin (40 mg/mL) were dissolved in CH_2_Cl_2_ by magnetic stirring to form an organic phase. PVA was dissolved in ultrapure water to form water phase (10 mg/mL). The organic phase was added dropwise over 10 min into the water phase under high shear emulsification (C25, ATS Engineering Ltd., Vancouver, Canada). CH_2_Cl_2_ was eliminated from the nanosuspension by evaporation at room temperature with magnetic stirring (1,000 rpm) overnight. The abamectin PLA nanospheres (Abam-PLA-NS) were collected by centrifuging the nanosuspension at 15,000 rpm for 10 min at 4 °C and the deposition was redispersed in deionized water; this process was repeated three times to remove as much surfactant as possible.

TA modified nanospheres were formulated by a self-assembly method. PEG (12 mg/mL) was added to the nanosuspension mentioned above, and followed by dropping TA (12 mg/mL). After stirring for 1h, the TA modified abamectin PLA nanospheres (Abam-PLA-Tannin-NS) were collected via centrifugation at 15,000 rpm for 10 min at 4 °C and was washed three times with deionized water.

### Characterization of nano-formulated abamectin

The hydrodynamic particle size and polydispersity index (PDI) of nanospheres were investigated at 25 °C by dynamic light scattering (DLS; Zetasizer Nano-ZS90, Malvern, Worcestershire, UK). The morphology of the nano-formulated abamectin was observed via transmission electron microscope (TEM, HT7700, Hitachi Ltd., Tokyo, Japan). About 6 μl of the dispersed nanospheres was dropped on the surface of a cleaned copper grid. The TEM images were performed at 80 kV and 10 mA after the nanospheres were completely dried. The abamectin loading efficiency of nanospheres was investigated by high performance liquid chromatography (HPLC, 1260 Infinity, Agilent Company, California, USA). In brief, an appropriate aliquot of nanospheres was dispersed in CH_2_Cl_2_ (5ml) and sonicated for 5min, followed by evaporation of the organic solvent at room temperature. Then abamectin was diluted to an appropriate volume with methanol. For the HPLC analysis, a C18 column (5 mm, 4.6 mm × 150 mm, Agilent Technologies; Santa Clara, CA, USA) was used to separate the target compound from others at room temperature. Methanol/water (90:10, v/v) was used as mobile phase at a flow rate of 1.0 mL/min. The wavelength of UV detector was 245 nm. Loading efficiency (%) = (weight of pesticide in nanospheres/weight of nanospheres) ×100%.

### Biological materials

For aphid rearing, broad beans (*Vicia faba* L.) were used as host plants. The seeds were sown in 30 cm × 20 cm boxes, which contained a 1:1 mixture of vermiculite and perlite. The plants were infested with aphids at two-leaf stage. Aphids *A*. *pisum* were collected from Gembloux, Belgium, and they had been reared in the laboratory for several years. Aphids were kept under controlled conditions (22 ± 2 °C, 70 ± 10% R.H. and 16L:8D of photoperiod).

First instars of lady beetles *A*. *bipunctata* were purchased from Biobest Group NV, Belgium. Larvae were reared on a diet of frozen *Ephestia kuehniella* (Lepidoptera, Pyralidae) eggs and water until pupation or death. All studies were conducted at 22 ± 1 °C, 30 ± 5% R.H., and a photoperiod of 16L:8D of photoperiod.

### Insecticidal effect of nano-formulated abamectin on the aphid *A*. *pisum*

An agar solution was prepared to perform the insecticidal assay on Petri dishes (diam. 3.5 cm) containing a broad bean leaf and aphids. Agar powder was mixed with distilled water (1%, w/w), heated until boiling and then allowed cooling while constantly mixing. After cooling for approximately 10 minutes, warm agar was poured into each Petri dish to a depth that was at least 3–4 mm. A round piece of leaf of 33 mm in diameter was cut using a sharpened metal tube, and put on the agar gel with abaxial surface facing skywards. Ten apterous aphid individuals were transferred onto each of the leaf discs using a fine brush.

The bioassay was conducted using Potter Precision Laboratory Spray Tower (Burkard Scientific, Uxbridge, UK) at a spray pressure 0.70 kg/cm^2^ (69 kPa; 10 psi) [33]. The biocidal efficacies of Abam-PLA-NS, Abam-PLA-Tannin-NS and the commercial EC were evaluated. Water was used as the untreated control. Based on preliminary experiments to establish the range of concentrations to be tested, six concentrations, 3.125 mg/L, 6.25 mg/L, 12.5 mg/L, 25 mg/L, 50 mg/L, and 100 mg/L were tested for each formulation. Each aphid-containing Petri dish was sprayed with 1 mL of the tested solution, representing a deposit of 27.9 ± 2.1 mg on the leaf-disc. Then, all dishes were sealed with a close-fitting, ventilated lids. Two days after the application, the number of alive aphids was counted in each dish. An aphid was considered dead if it failed to react when touched by the brush. There were 3 replicates in each treatment (formulation × concentration) and for the water control.

### Non-target effect of nano-formulated abamectin on coccinellid predator *A*. *bipunctata*

Abamectin was relatively safe to adult lady beetles [34–35], so first instars were selected as the objects in this study. Larvae were treated with Abam-PLA-NS, Abam-PLA-Tannin-NS and the commercial EC of abamectin, using the same six concentrations in the aphid bioassay. Controls were maintained using water alone. Abamectin acts on glutamate-gated chloride ion channels in arthropods to produce long-term, high-intensity inhibitory effects, causing insects to die. Abamectin has oral and contact toxicity. Therefore, three groups of insecticidal assays were conducted on lady beetles or aphids with Potter spray tower:

**(a) Topical exposure**: Ten larvae were transferred to a Petri dish and then 1 mL of the tested solution was sprayed. Larvae were then individually transferred to clean plastic Petri dishes and checked for mortality daily.
**(b) Residual exposure**: Petri dishes were sprayed with 1mL of the tested solution (n = 10 for each concentration of a tested solution). A single larva was then individually transferred to a Petri dish. After 24 h of contact, they were transferred to clean plastic Petri dishes and checked for mortality daily.
**(c) Oral exposure**: 20 aphids (*A*. *pisum)* were sprayed with 1mL of the tested solution. After air dry, they were transferred to a clean plastic pot where a lady beetle larva was introduced (n = 10 for each concentration of a tested solution). Frozen *Ephestia kuehniella* eggs and water were offered after all the aphids died. Larvae were checked for mortality daily.

### Statistical analysis

Insect mortality was corrected by Abbott’s formula [36], taking into account the natural mortality observed on the control. The mortality of insect exposure to different formulations was analyzed by one-way ANOVA. Probit analysis was performed in order to estimate the LC_50_ [37], in which, a Chi-square Goodness-of-fit test was used to analyze the mortality data. The dose-mortality relationships were considered valid when the observed data and the expected data did not diverge significantly (P<0.05). Data analysis was carried out on SPSS Statistics V.17 (IBM).

## Results and discussions

### Characterization of nano-formulated abamectin

**Fig 1:**
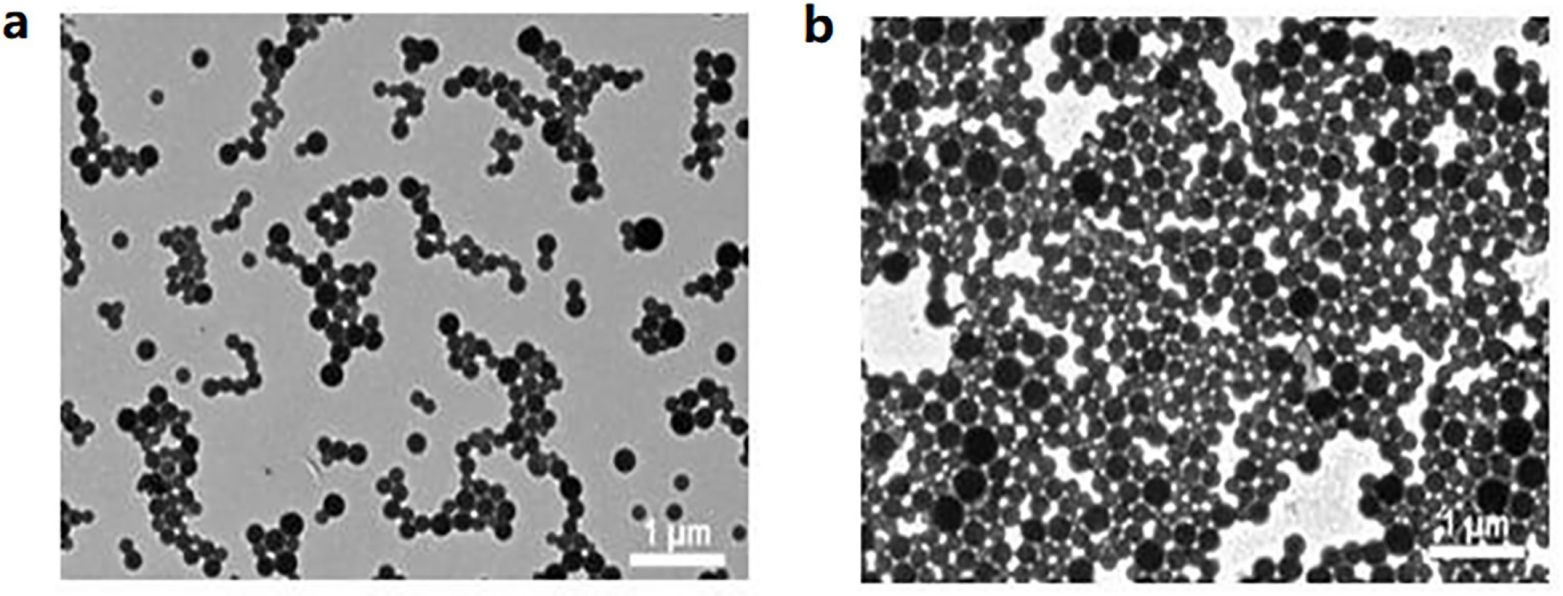
TEM images of Abam-PLA nanoparticles (a) and Abam-PLA-Tannin nanoparticles (b).

The hydrodynamic size of Abam-PLA-NS measured by DLS was 240.7±1.9 nm, and it increased to 243.6±1.2 nm for the Abam-PLA-Tannin-NS. The PDIs were 0.03±0.02 and 0.02±0.01 for Abam-PLA-NS and Abam-PLA-Tannin-NS, respectively. The low PDIs implied a narrow size distribution and monodispersion, which was also confirmed by TEM characterization results. TEM imaging (Fig 1) indicated that these nanoparticles exhibited nearly uniform spheres, and the statistical average size of 100 nanoparticles from the TEM images was 150.7±2.2 nm for Abam-PLA-NS, and it increased to 156.5±2.4 nm for the Abam-PLA-Tannin-NS. These results suggested that the surface of Abam-PLA-NS was capped with TA to form Abam-PLA-Tannin-NS. According to the HPLC analytical results, the abamectin loading efficiency of Abam-PLA-NS was 46.9%, and it decreased to 38.9% for Abam-PLA-Tannin-NS.

### Insecticidal effect of nano-formulated abamectin on the aphid *A*. *pisum*

The bioassay results of nano-formulations and commercial EC on *A*. *pisum* were showed in Table 1. The data fitted the linear model (no statistically significant deviation of data from the regression equation). LC_50_ values were 33.3, 10.1 and 13.1 mg/L for Abam-PLA-NS, Abam-PLA-Tannin-NS, and abamectin EC, respectively. However, there was no significant difference among the three formulations, because their 95% confidence limits overlapped. And the statistical results of mortality also confirmed this (F = 0.949, P = 0.409).

**Table 1:** Laboratory bioassay results of abamectin formulations against aphids after 48h.

Formulation	Toxicity regression equation	R^2^	LC_50_ (mg/L)	95% confidence limit	χ^2^ (df = 4)	P
**Abam-PLA-NS**	y = 2.87+1.40x	0.886	33.3	18.6–59.5	1.296	0.862
**Abam-PLA-Tannin-NS**	y = 4.11+0.88x	0.812	10.1	4.2–23.9	4.336	0.362
**Abmectin EC**	y = 3.38+1.45x	0.987	13.1	7.5–22.8	1.679	0.795

Nano-formulated pesticides are expected to improve the efficiency of pesticide and reduce environmental pollution [6, 10]. These two solvent-free nano-formulated abamectin exhibited similar biocidal efficacy on aphids, because nano-sized formulations can improve the adhesivity and penetrability of pesticides on surface of organisms [20, 38]. Abam-PLA-Tannin-NS had a lower LC_50_ than Abam-PLA-NS, which attributed to the enhancing contact of abamectin on the epidermis of aphids, due to the adhesive properties of TA.

Abamectin is categorized as highly toxic with acute oral and dermal toxicity with low LD_50_ concentrations for different organisms [39]. However, its popularity is growing due to the effective pest control. Usually, in the bioassay, the type, developmental stage and physiological condition of the target organism have a great influence on the efficacy of the pesticide. In addition, the environmental condition may also affect the response of the target to the agent. The recorded LC_50_ of abamectin for mustard aphid *Lipaphis erysimi* Kalt (Hemiptera: Aphididae) was 0.63 mg/L [40]. The recorded LC_50_ for peach aphid *Myzus persicae* Sulzer (Hemiptera: Aphididae) were 1.5 mg/L [41], and that was 5.5 mg/L for Anjum et al’s bioassay [42]. But results of our study could get support from our recent bioassay on *M*. *persicae* (LC_50_ of 17.38 mg/L and 10.68 mg/L for Abam-PLA-NS and Abam-PLA-Tanning-NS respctively) [32].

### Non-target effect of nano-formulated abamectin on coccinellid predator *A*. *bipunctata*

Some studies have confirmed that nanoformulations were harmless to non-target organism, such as different cell lines and soil microorganisms [43], but only a few safety studies were carried out on natural predators [44].

The biocidal effects of all tested formulations against lady beetle larvae after 48 h and 120 h are presented in Tables 2, 3 and 4 for topical exposure, residual exposure and oral exposure, respectively. For all the trials, there was no statistically significant deviation of data from the regression equation. For each exposure group, overlapped 95% confidence limits indicated non-significant difference among three formulations. There was no significant difference of larvae mortality in each group as well (topical exposure, 48 h, F = 0.024, P = 0.976; 120 h, F = 0.118, P = 0.889; residual exposure 48 h, F = 0.658, P = 0.532; 120 h, F = 0.317, P = 0.733; oral exposure, 48 h, F = 0.139, P = 0.872; 120 h, F = 0.056, P = 0.946).

**Table 2:** Topical exposure for three abamectin formulations on lady beetle larvae.

Time	Formulation	Toxicity regression equation	R^2^	LC_50_ (mg/L)	95% confidence limit	χ^2^ (df = 4)	P
**48 h**	**Abam-PLA-NS**	y = 3.79+1.10x	0.871	12.5	6.2–25.2	5.817	0.121
	**Abam-PLA-Tannin-NS**	y = 3.44+1.26x	0.789	16.6	8.9–31.0	9.005	0.061
	**Abmectin EC**	y = 2.72+1.75x	0.935	19.4	12.0–31.3	4.626	0.328
**120 h**	**Abam-PLA-NS**	y = 3.75+1.44x	0.844	7.4	4.2–13.2	1.174	0.882
	**Abam-PLA-Tannin-NS**	y = 3.92+1.37x	0.831	6.0	3.3–11.0	1.945	0.746
	**Abmectin EC**	y = 3.36+1.64x	0.877	10.3	6.2–17.1	1.105	0.893

**Table 3:** Residual exposure for three abamectin formulations on lady beetle larvae.

Time	Formulation	Toxicity regression equation	R^2^	LC_50_ (mg/L)	95% confidence limit	χ^2^ (df = 4)	P
**48 h**	**Abam-PLA-NS**	y = 2.47+2.26x	0.976	13.2	8.8–19.7	4.808	0.308
	**Abam-PLA-Tannin-NS**	y = 3.52+1.27x	0.983	14.7	7.9–27.3	3.395	0.494
	**Abmectin EC**	y = 3.72+0.66x	0.846	83.9	23.5–299.8	3.162	0.531
**120 h**	**Abam-PLA-NS**	y = 3.03+2.01x	0.920	9.7	6.2–15.0	7.926	0.094
	**Abam-PLA-Tannin-NS**	y = 3.40+1.76x	0.960	8.1	5.0–13.6	4.303	0.367
	**Abmectin EC**	y = 3.14+1.58x	0.805	15.3	9.1–25.6	2.376	0.667

**Table 4:** Oral exposure for three abamectin formulations on lady beetle larvae.

Time	Formulation	Toxicity regression equation	R^2^	LC_50_ (mg/L)	95% confidence limit	χ^2^ (df = 4)	P
**48 h**	**Abam-PLA-NS**	y = 2.57+1.16x	0.884	110.2	50.9–238.6	4.935	0.294
	**Abam-PLA-Tannin-NS**	y = 1.63+1.82x	0.998	71.3	42.2–120.4	5.414	0.247
	**Abmectin EC**	y = 1.85+1.95x	0.806	41.9	26.4–66.4	2.770	0.597
**120 h**	**Abam-PLA-NS**	y = 2.98+1.09x	0.816	74.0	34.7–157.5	4.354	0.360
	**Abam-PLA-Tannin-NS**	y = 1.13+2.58x	0.984	31.3	21.6–45.6	4.531	0.339
	**Abmectin EC**	y = 3.01+1.46x	0.762	22.5	13.0–39.2	4.899	0.298

Taking LC_50_ as the toxicological endpoint, two nanoformulations showed higher contact toxicity than EC in both topical and residual applications 48 h and 120 h after the spray. It resulted in a similar trend as compared to aphids. This suggested that the nano-formulated abamectin had no selectivity for either insect group. For the commercial formulation, free abamectin on the surface of insects and Petri dishes were rapidly degraded, thereby reducing exposure to toxic residues. Nanoformualtions could enhance the stability of the pesticide and inhibit the degradation of abamectin [32, 45], then, improved the toxicity to ladybeetles. While the oral exposure results showed totally opposite trends. Both nano-formulated abamectin showed higher LC_50_ than abamectin EC after feeding. It could be accounted by the fact that PLA prevented abamectin from contacting with the lady beetles after entering the digestive tract [46]. The existence of TA also increased the toxicity of abamectin nanospheres due to the improved adhesion.

When applied in crop protection, abamectin turns into a source of concern for non-targeted beneficial arthropods and evolving resistance in pests [47]. The effects of 13 agrochemicals used in grapevines on spider mite *Panonychus ulmi* (Acari: Tetranychidae) and the predatory mite *Neoseiulus californicus* (Acari: Phytoseiidae) were evaluated, and exposure to abamectin significantly reduced survival both of *P*. *ulmi* and of *N*. *californicus* [48]. A study proved that commercial abamectin was the most harmful substance (in term of lethal and sublethal effects) among 14 tested pesticides against *Orius laevigatus* Fieber (Hemiptera: Anthocoridae), a commonly used predator in biological control programs [49]. It was found that commercial abamectin EC at recommended concentration combined with 0.5% summer oil was highly toxic to *Rhyzobius lophanthae* Blaisdell (Coleoptera: Coccinellidae) adults and larvae in both direct applications and pesticide residue situations [50]. The recorded LC_50_ of abamectin for the first instar and adult of multicolored Asian lady beetles, *Harmonia axyridis* (Coleoptera: Coccinellidae) were <0.09 mg/L and 4.88 mg/L, respectively, with a topical method in laboratory condition [51]. Whereas abamectin commercial formulation of 18 mg/L could cause 14.66% mortality of *H*. *axyridis* in eggplant ecosystem [52] with the spray method. It is difficult to verify such contradictory results, which may partly attribute to the differences of both experimental methods and environmental conditions. Compared the topical exposure results with the recorded LC_50_, two solvent-free nanoformulations were more safety to ladybeetles.

## Conclusions

In this study, two solvent-free nano-formulated abamectin were prepared, and their biocidal efficacy on aphids and on lady beetles was tested. Generally, there was no significant difference between nanoformulations and commercial EC in mortality of the acute toxicity. The environment-friendly compositions of nanoformulations make them more suitable as the plant protection products in IPM strategies. It is noticeable that the side effects on the non-target organisms at different sublethal concentrations, should also be considered for a complete understand of the nanoformulations [29, 53]. Meanwhile, further studies on the field trial would be certainly worthwhile.
